# When Lead Goes to Your Head: Genotype May Link Exposure and Meningioma

**Published:** 2005-09

**Authors:** Michael Szpir

Scientists know very little about the causes of most brain tumors. A small percentage of cases can be explained by familial syndromes, or by exposure to ionizing radiation, but the precise roles of specific genes or other environmental factors, such as lead, remain largely unexplored. A research team now reports an association between a genetic variant for δ-aminolevulinic acid dehydratase (ALAD)—an enzyme involved in the synthesis of heme—and an increased risk of developing meningioma, a tumor that occurs in the membranes covering the brain and the spinal cord **[*EHP* 113:1209–1211]**.

Some previous studies suggested that people who carry an *ALAD* polymorphism known as *ALAD2* tend to have higher concentrations of lead in their blood. Other research has indicated that occupational exposure to lead may increase the risk of meningioma. The findings of the current study suggest a possible link between these two results.

The team discovered a connection between *ALAD2* and meningioma in a study of 573 patients with brain tumors from hospitals in Arizona, Massachusetts, and Pennsylvania. The patients were compared to 505 control subjects who were admitted to the same hospitals for conditions that did not involve tumors. Of the brain tumor patients, 151 had meningioma, 355 had glioma (a cancer that grows from glial cells in the brain), and 67 had acoustic neuroma (a tumor of the auditory nerve).

The *ALAD* genotype—based on the *ALAD1* and *ALAD2* alleles—was determined for each patient and each control subject. Possible links between the *ALAD2* allele and the brain tumors were investigated using unconditional logistic regression.

The statistical analyses revealed that people who carried the *ALAD2* allele (heterozygotes and homozygotes) were 1.6 times more likely than the *ALAD1* homozygotes to develop meningioma. This modest but significant association was stronger in males, who were 3.5 times more likely to develop meningioma if they had the variant allele. However, the authors caution that their sample size may be too small to draw conclusions about gender-related effects. They saw no increased risk linked with the *ALAD2* allele for glioma or acoustic neuroma.

These results raise the question of how the *ALAD2* allele might increase the risk of meningioma. Previous work by the same team, based on the same study subjects, found an elevated risk of meningioma for occupational groups that may be exposed to lead, including auto body painters and industrial production supervisors. Certain other studies have shown that individuals who carry the *ALAD2* allele have higher levels of lead in their blood. Together, these results suggest that lead may play a role in the link between the *ALAD2* allele and meningioma. The researchers recommend that future investigations should consider the combined effects of exposure to lead and the *ALAD2* allele on the incidence of this cancer.

## Figures and Tables

**Figure:**
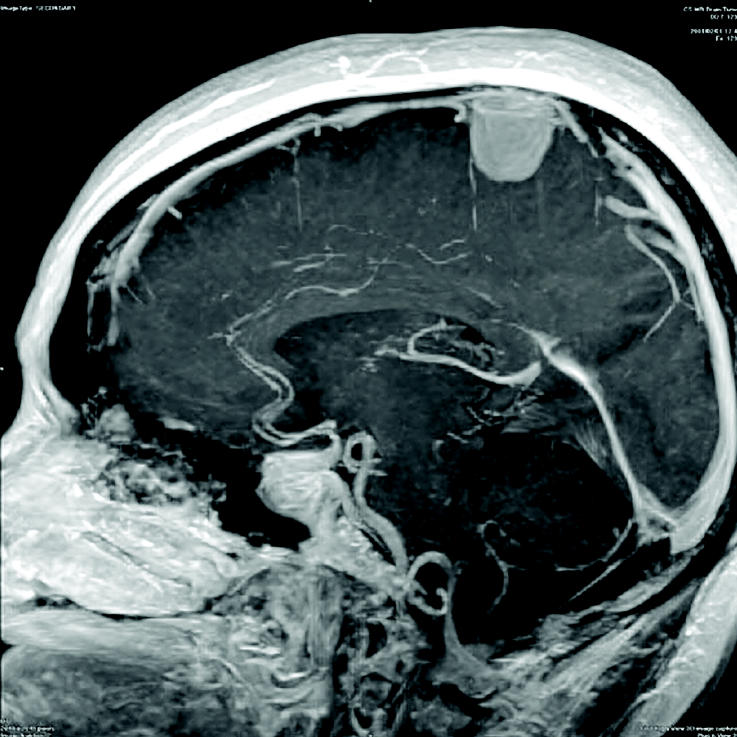
Chasing leads on brain tumors. Information on what causes brain tumors is fragmented; however, new data may tie together clues about lead exposure and a predisposition to develop meningioma.

